# Women’s empowerment and child nutrition in a context of shifting livelihoods in Eastern Oromia, Ethiopia

**DOI:** 10.3389/fnut.2023.1048532

**Published:** 2023-06-29

**Authors:** Karah Mechlowitz, Nitya Singh, Xiaolong Li, Dehao Chen, Yang Yang, Anna Rabil, Adriana Joy Cheraso, Ibsa Abdusemed Ahmed, Jafer Kedir Amin, Wondwossen A. Gebreyes, Jemal Y. Hassen, Abdulmuen Mohammed Ibrahim, Mark J. Manary, Gireesh Rajashekara, Kedir Teji Roba, Ibsa Aliyi Usmane, Arie H. Havelaar, Sarah L. McKune

**Affiliations:** ^1^Department of Social and Behavioral Sciences, College of Public Health and Health Professions, University of Florida, Gainesville, FL, United States; ^2^Department of Animal Sciences, Institute of Food and Agricultural Sciences, University of Florida, Gainesville, FL, United States; ^3^Emerging Pathogens Institute, University of Florida, Gainesville, FL, United States; ^4^Food Systems Institute, University of Florida, Gainesville, FL, United States; ^5^Department of Environmental and Global Health, College of Public Health and Health Professions, University of Florida, Gainesville, FL, United States; ^6^Department of Biostatistics, College of Public Health and Health Professions, University of Florida, Gainesville, FL, United States; ^7^Yale School of Public Health, Yale University, New Haven, CT, United States; ^8^Department of Public Health and Health Policy, College of Health and Medical Sciences, Haramaya University, Haramaya, Ethiopia; ^9^School of Public Health, Haramaya University, Haramaya, Ethiopia; ^10^College of Veterinary Medicine, Haramaya University, Haramaya, Ethiopia; ^11^Global One Health Initiative, College of Veterinary Medicine, The Ohio State University, Columbus, OH, United States; ^12^School of Rural Development and Agricultural Innovation, College of Agriculture and Environmental Science, Haramaya University, Haramaya, Ethiopia; ^13^Office of Research Affairs, Haramaya University, Haramaya, Ethiopia; ^14^Department of Pediatrics, Washington University, St. Louis, MO, United States; ^15^Center for Food Animal Health, Department of Animal Sciences, College of Food, Agricultural, and Environmental Sciences, The Ohio State University, Wooster, OH, United States; ^16^Center for African Studies, University of Florida, Gainesville, FL, United States

**Keywords:** women’s empowerment, child nutrition, livelihood, livestock production, khat

## Abstract

Agriculture, and particularly livestock and animal source foods, has been closely linked to improvements in human nutrition. Production, income, and women’s empowerment improve household food security and child nutritional outcomes in interacting ways. Khat production in Eastern Ethiopia is changing the economic and livelihood landscape for communities that have traditionally relied upon small-scale mixed agriculture and livestock production. How this shifting livelihood landscape and the empowerment of women in these communities are affecting nutritional outcomes has not been investigated. Using cross-sectional data collected during formative research for the Campylobacter Genomics and Environmental Enteric Dysfunction (CAGED) project, we developed models to examine the roles of livelihood activities, including livestock production, staple crop production, and khat production, and women’s empowerment in child nutrition outcomes. Survey participants were randomly selected mothers of children aged 10–15 months from Haramaya district, Eastern Hararghe, Oromia, Ethiopia. Nested logistic regression models were performed for each nutrition outcome: children’s animal source food consumption, children’s dietary diversity, and child stunting, wasting, and underweight. Explanatory variables included those for livelihood (tropical livestock unit, crop production, and khat production ladder) and women’s empowerment (as indicated by domains of the Women’s Empowerment in Agriculture Index), and covariates including child sex, mother’s age, mother’s education, assets, income, and kebele. Results indicated that khat production and tropical livestock units were not significantly associated with any of the child nutrition outcomes. However, results did indicate that the odds of reporting child animal source food consumption in households where the mother was empowered in the leadership domain was 3.33 times that in households where the mother wasn’t (*p* < 0.05). In addition, the odds of having a stunted child in households where the mother was empowered in the time domain was 2.68 times that in households where the mother wasn’t (*p* < 0.05). The results from this study both support and complicate the existing literature on the associations between women’s empowerment in agriculture and child nutrition outcomes, underscoring the important role that livelihood, contextual factors, and location may have on the complex relationship between empowerment domains and nutritional outcomes.

## 1. Introduction

Agriculture contributes to nutrition through multiple, increasingly well-established pathways, including production, income, and women’s empowerment (1). In sub-Saharan Africa (SSA) where the livelihoods of many smallholder farming households are dependent on a mix of staple crop, cash crop, and/or livestock production, the health of humans and livestock are intrinsically linked. Livestock, in particular, have been recognized as playing a critical role in the health and livelihoods of smallholder households in SSA (2). The specific role of livestock production and animal source food production and consumption on the nutritional outcomes of smallholder farmers indicates an overall benefit, especially when women own the livestock or are decision-makers. Production of animal source foods (ASFs) for the household’s own consumption is the most direct pathway by which increased livestock production can lead to greater food security at the household level, contributing to improved household nutrition (3). Animal source foods provide high-quality protein and essential micronutrients that can improve the nutritional status of children and promote growth and cognitive development. Interventions investigating the impact of a livestock production intervention on ASF consumption have significantly increased ASF consumption among children in Burkina Faso, Ethiopia, and Kenya (4–6). Income from livestock production is another way through which smallholder households may benefit, as livestock and livestock products can be sold to produce cash income and can also be used for draft power and transport (7). As smallholder households adapt to changing environmental and market conditions, production for sale often occurs alongside production for consumption (3).

Women’s empowerment, the third pathway linking production to nutrition, is considered a necessary means to achieving goals of development (8). Women in smallholder households generally play a crucial role in caring for and managing livestock and crop production and are, as such, key actors in food systems. Empowering women in agriculture by giving them ownership of their assets and power in decision-making has the capacity to not only close gender gaps and improve the economy (9, 10), but also to strengthen food security at the household level and allow for improved health outcomes for both women and children (11, 12). Research has delineated a positive relationship between livestock ownership and improved nutrition, as well as a link between women’s ownership of assets and household food security (13, 14). When development programs invest in women, outcomes can be more readily achieved through increasing earning potential, more control over key resources, and more decision-making autonomy (8, 15). Similarly, when women are empowered, they are better equipped to sustain their children’s health as well as their own, and they are more productive in agriculture (16).

In Eastern Ethiopia, a region characterized by widespread poverty and high rates of childhood malnutrition, most smallholder households rely on a mix of livestock and crop production for their livelihoods. However, the agricultural backdrop of parts of the Oromia region in eastern Ethiopia has shifted in recent years to include extensive production of khat – a mild stimulant grown as a cash crop (17). Khat production has become increasingly commercialized in eastern Ethiopia, shifting smallholder farmers away from food-based crop production towards cash crop production (18, 19). Between 2003 and 2017, khat production in Harari and Oromia regions increased by 140 and 306%, respectively (20). Khat is a dominant cash crop in the region that can be harvested year-round. Its economic importance to smallholder farmers in rural eastern Ethiopia has grown to become a major source of regular cash income (18, 21, 22). Income per hectare from khat among smallholder farmers in Ethiopia has been estimated to be 14.5 times higher than that from grain/cereals, 17 times higher than that from pulses, 6 times higher than that from oilseeds, and 4 times more than from coffee, surpassing many major agricultural crops by several margins (21). In Haramaya district of the Oromia region, smallholder households often practice horticulture crop production alongside khat production to simultaneously generate produce for food and for cash (21). While khat can provide important economic benefits to households, there are some concerns regarding khat consumption that need to be acknowledged. The implications of khat consumption on health, particularly for pregnant women, is understudied. However, some research has demonstrated that consuming khat may suppress appetite (23), and a recent study found that pregnant women who reported chewing khat were only half as likely to consume ASF as those who do not (24).

At major markets in eastern Ethiopia khat tends to be sold largely by women (25). Women’s participation in these khat markets may provide them with greater control over income generated from the sale of khat and may reduce their workload, which are important aspects of women’s empowerment. Furthermore, the income generated from the sale of khat and women’s participation in trade may facilitate access to markets and foods, contributing to a more diverse diet and improved nutritional outcomes, particularly for children. However, there is little research investigating women’s empowerment in the context of rapidly transitioning livelihoods – such as the transition occurring in parts of eastern Ethiopia from subsistence farming to khat production – and the implications this may have on nutritional outcomes for children.

Within the shifting livelihood composition described above, this study aims to examine associations between women’s empowerment and nutritional outcomes of young children in Haramaya, Ethiopia. Specifically, we will investigate the relationship between livelihood activities, including livestock production, staple crop production, and khat production, and women’s empowerment on child nutrition outcomes (including child ASF consumption, child dietary diversity, stunting, wasting, and underweight) in eastern Ethiopia.

## 2. Materials and methods

This study analyzed data collected from the Campylobacter Genomics and Environmental Enteric Dysfunction (CAGED) formative research, a community-based, cross-sectional study. The CAGED project was designed to assess the prevalence of stunting, environmental enteric dysfunction (EED), and Campylobacter colonization in young children and to characterize the sociodemographic background in rural eastern Ethiopia. Study aims, research questions, and detailed methodology are published elsewhere (18, 26, 27). The study methodology is briefly described below.

### 2.1. Study area

Haramaya district is a semi-arid ecological zone in the eastern region of Ethiopia where households rely heavily on livestock and crop production for their livelihoods. Farmers in the area produce mostly khat, maize, and sorghum (22). Haramaya woreda consists of 33 kebeles, 12 of which are covered by the Haramaya Health Demographic Surveillance and Health Research Center (HDS-HRC). The district is characterized by widespread poverty, youth unemployment, low literacy, and high fertility. The study area has high childhood malnutrition rates, particularly stunting, which contributed to its selection for this research. Haramaya is also particularly affected by climate change. Increasingly erratic rainfall, reduced ground water availability, and land degradation are threatening the livelihoods of many households in the district. Women are primarily responsible for taking care of infants and livestock (18, 22). This district is also a major khat-growing area, with several major towns where khat is sold to local consumers. In local communities, khat is largely sold through informal channels, but the district also houses khat export centers, most notably Aweday, as well as khat transport routes, where khat is sold as wholesale to be transported to other regions of Ethiopia and exported to other countries (21, 28).

### 2.2. Design and sample

To maximize geographic distance between kebeles, 5 out of the 12 kebeles covered by the HDS-HRC were selected for the formative research. Households in the 5 kebeles were randomly selected to complete household surveys. Inclusion criteria of the households included the presence of at least 3 chickens in the homestead (defined as the small collection of households that are physically connected to one another, often housing an extended family) and willingness to participate in and conform to the study requirements. Exclusion criteria of the households included families that were participating in another animal husbandry project, not residing in Haramaya woreda for at least 3 months, or had a mother who did not live in Haramaya woreda when the child was born. Eligibility criteria of the child included the child’s mother being the caretaker and the child being 11–13 months when consent was given. The child was excluded from the study if she/he presented with a visible congenital abnormality or a serious medical illness, and if the child or her/his mother required an extended stay in hospital after birth. Data collection occurred between September and December 2018. A total of 102 households with children aged 11–13 months were randomly selected to participate and completed the household survey.

### 2.3. Data collection

The household questionnaire was developed collaboratively by University of Florida (UF) and Haramaya University (HU) team members. Following several rounds of revisions with social scientists and field workers, the questionnaire was finalized to include culturally appropriate language and answer choices. The questionnaire was translated to Afaan Oromo and administered to participants by bilingual, trained data collectors. Data were collected and managed using REDCap software on Samsung Galaxy tablets (29, 30). Questions included topics of demographics, livelihoods, wealth, animal ownership, animal management and disease, water, sanitation and hygiene, health, nutrition, and women’s empowerment. The same team also collected child stool and urine samples, animal fecal samples, and data on anthropometric measurements of children, which have been described elsewhere (18, 26, 27).

### 2.4. Data

#### 2.4.1. Variables

Primary outcome variables of interest in this study were children’s ASF consumption, children’s dietary diversity, stunting, wasting, and underweight. To strengthen the robustness of the models, continuous and ordinal variables were dichotomized by medians or generally accepted cut-offs. Children’s ASF consumption was coded as 0/1, indicating whether or not a child has consumed ASF. Dietary diversity was dichotomized into insufficient or sufficient dietary diversity based on having a dietary diversity score of less than or greater than or equal to five. Stunting, wasting, and underweight were based on having a height-for-age *z*-score (HAZ), a weight-for-length *z*-score (WLZ), and a weight-for-age *z*-score (WAZ) of less than −2, respectively (31).

The explanatory variables included tropical livestock unit (TLU), crop production, khat production ladder, and women’s empowerment. The TLU is a reference unit that allows for the aggregation of various livestock species and is commonly used in low- and middle-income countries as an indicator of food security risk (32). Tropical livestock units were coded according to Jahnke and then dichotomized into low or high TLUs based on having TLU less than or greater than or equal to the median (33). Crop production was coded 0/1 based on households’ report of having crop production as its dominant livelihood activity. The khat production ladder was constructed to represent level of involvement in khat production, as reported by the woman. The survey questions used to construct this variable included (1) khat produced for livelihood, (2) khat production as primary produce for livelihood, and (3) khat production supported by irrigation. Households could score between zero to four for this khat production ladder variable. If a household did not produce khat for livelihood, did not report khat as primary produce for livelihood, and did not use irrigation to support khat production, then they received a score of zero, indicating no involvement in khat production. A score of one, two, or three indicated that a household was involved in one, two, or all three khat production activities, respectively. Thus, a higher score indicated greater involvement in khat production. The khat production ladder variable was collapsed into two categories: no to little involvement in khat production and medium to high involvement in khat production.

Women’s empowerment scores were calculated employing the Women’s Empowerment in Agriculture Index collected for women in surveyed households (A-WEAI) (34). Empowerment adequacy scores (which were binary 1/0) were calculated for six variables represented under 5 domains, namely, production, resource (including ownership and credit variables), income, leadership and time. Weighted scores as defined by A-WEAI scoring scheme (34) were summed up to calculate aggregated overall women empowerment score (binarized with cutoff >0.8 as empowered).

Other covariates potentially associated with the outcome variables were selected based on *a priori* knowledge established in the literature, including child sex, mother’s age, mother’s education, assets, income, and kebele. Mother’s age was dichotomized by median into younger or older age group. Mother’s education was based on literacy, indicating mother’s (in)ability to read and write. The asset variable was constructed by summing the presence and absence of items owned by the household to create counts of assets of each household. The crude counts were then assigned into quartiles. The income variable was constructed by adding income from different household activities (e.g., livestock and crops). Total income was then split into quartiles to indicate an income score of 1 to 4. Finally, the first and last 2 levels of each variable were combined to indicate low/high assets or income.

Given established important differences in the five kebeles, the kebele variable was recoded from a five-item categorical variable to a dichotomized variable, which was recoded for each model, based on the outcome variable of interest. For example, in models examining the outcome of dietary diversity, the five kebeles were divided into two categories based on the prevalence of sufficient dietary diversity; consequently, kebeles two and four were combined, and kebeles one, three, and five were combined for the dietary diversity models. For the outcome of ASF consumption, the kebeles were categorized into two categories based on the prevalence of ASF consumption, and so forth.

#### 2.4.2. Analysis

Data analysis was performed in R statistical software version (35). Descriptive analyses were performed to explore sociodemographic characteristics of the study population. Bivariate analyses were conducted to screen the association between each explanatory variable and each nutrition outcome. As all variables are categorical or ordinal, the chi-squared test was performed when all cells in the contingency table were at least five; Fisher’s exact test was used otherwise. Multivariable logistic regression models were performed for each nutrition outcome, where covariates with *p*-values <0.2 from the bivariate analyses were used to build nested models. Explanatory variables of primary interest (TLU, khat production ladder, crop production as dominant livelihood, and overall women’s empowerment) were included in the models for the outcomes of child ASF consumption, stunting, and underweight, regardless of their statistical significance in the bivariate analyses. Overall empowerment was expanded into the five individual domains of empowerment (production, resource credit, income, leadership, and time) for the outcomes of child ASF consumption, stunting, and underweight. For the outcome of dietary diversity, because of insufficient numbers in contingency tables between the explanatory variables and dietary diversity, only descriptive data and bivariate analyses are presented. For the outcome of wasting, because of a low prevalence of wasting in the study sample, only descriptive data are presented. For the outcome of underweight, crop production as dominant livelihood was removed due to insufficient numbers in contingency tables. Multicollinearity was checked using variance inflation factor, and observations with missing values were excluded from the analyses.

## 3. Results

### 3.1. Descriptive data

**Figure 1:**
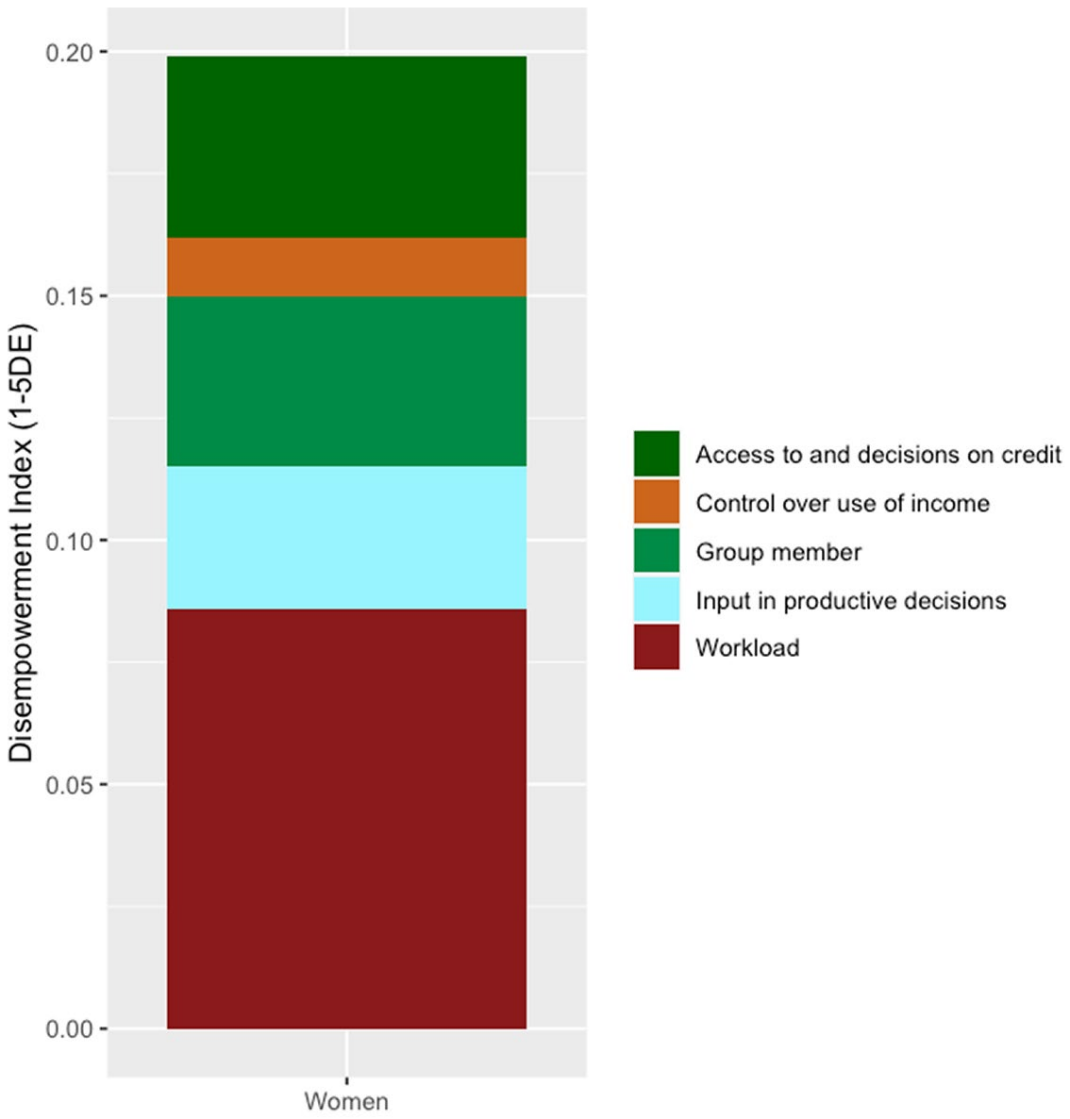
Contributions of each indicator to women’s disempowerment. ^*^The indicator ownership of assets had no contribution to women’s disempowerment.

Descriptive data are displayed in Table 1. These data were tabulated to display the presence of each variable within each outcome. The median TLU was 1.2. Of those households with high TLU (above the median), 39.3% reported no child ASF consumption. Insufficient child dietary diversity was high (85.3%), with 47.1% of households reporting insufficient child dietary diversity also reporting no child ASF consumption. The median maternal age was 26 years, with 85.1% of households with older mothers (age above the median) reporting insufficient child dietary diversity. Only 12.7% of households reported crop production as their dominant livelihood. The majority of households were involved in khat production, with 36.3% having a moderate level of involvement in khat production. Stunting, wasting, underweight, insufficient child dietary diversity, and lack of child ASF consumption was most prevalent in kebele one. Figure 1 displays the contributions of each indicator of the A-WEAI to women’s disempowerment. This figure illustrates that the indicator contributing most to women’s disempowerment was workload, while ownership of assets had no contribution to women’s disempowerment. Besides ownership of assets, the indicator contributing least to disempowerment was control over use of income.

**Table 1:** Characteristics of participating households and children.

Household characteristics^†^	Stunted *N* (%)	Wasting *N* (%)	Underweight *N* (%)	Insufficient DD *N* (%)	No child ASF consumption *N* (%)	Total *N* (%)
High TLU	21 (37.5)	2 (3.6)	15 (26.8)	47 (85.5)	22 (39.3)	56 (54.9)
High assets	18 (35.3)	3 (5.9)	12 (23.5)	42 (89.4)	19 (37.3)	51 (50.0)
High income	21 (41.2)	3 (5.9)	12 (23.5)	41 (89.1)	18 (35.3)	51 (50.0)
Maternal age above median	20 (39.2)	3 (5.9)	15 (29.4)	40 (85.1)	21 (41.2)	51 (50.0)
Maternal education (able to read/write)	8 (30.8)	0 (0)	5 (19.2)	23 (88.5)	12 (4.6)	26 (25.5)
Kebele number						
Kebele 1	16 (51.6)	3 (9.7)	11 (35.5)	28 (100.0)	20 (64.5)	31 (30.4)
Kebele 2	4 (36.4)	1 (9.1)	2 (18.2)	9 (81.8)	3 (27.3)	11 (10.8)
Kebele 3	7 (35.0)	0 (0)	5 (25.0)	19 (100.0)	10 (50.0)	20 (19.6)
Kebele 4	4 (40.0)	1 (10.0)	4 (40.0)	7 (70.0)	1 (10.0)	10 (9.8)
Kebele 5	11 (36.7)	0 (0)	5 (16.7)	24 (85.7)	11 (36.7)	30 (29.4)
Crop production as primary livelihood	7 (53.8)	2 (15.4)	3 (23.1)	9 (75.0)	4 (30.8)	13 (12.7)
Khat production ladder						
No khat	4 (44.4)	1 (11.1)	3 (33.3)	9 (100.0)	3 (33.3)	9 (8.8)
Low	9 (31.0)	0 (0)	6 (20.7)	22 (78.6)	10 (34.5)	29 (28.4)
Medium	16 (43.2)	2 (5.4)	11 (29.7)	32 (94.1)	19 (51.4)	37 (36.3)
High	13 (48.1)	2 (7.4)	7 (25.9)	24 (96.0)	13 (48.1)	27 (26.5)
WEAI Domains						
Production	34 (40.0)	4 (4.7)	23 (27.1)	72 (90.0)	38 (44.7)	85 (83.3)
Resource ownership	41 (100.0)	5 (100.0)	26 (100.0)	86 (100.0)	43 (100.0)	100 (98.0)
Resource credit	3 (42.9)	0 (0)	2 (28.6)	7 (100.0)	4 (57.1)	7 (6.9)
Income	38 (40.4)	4 (4.3)	24 (25.5)	80 (89.8)	41 (43.6)	94 (92.2)
Leadership	34 (41.5)	5 (6.1)	21 (25.6)	70 (90.9)	33 (40.2)	82 (80.4)
Time	28 (50.9)	3 (54.5)	17 (30.9)	45 (88.2)	23 (41.8)	55 (53.9)
Overall empowerment	20 (46.5)	2 (4.7)	12 (27.9)	35 (87.5)	19 (44.2)	43 (42.2)
Child Characteristics						
Insufficient dietary diversity	37 (42.5)	4 (4.6)	21 (24.1)	–	41 (47.1)	87 (85.3)
Sex, female	24 (46.2)	2 (4.0)	15 (30.6)	45 (91.8)	22 (42.3)	52 (51.0)

Cells display *N* (% of outcome as proportion of household or child characteristic).

### 3.2. Bivariate analysis

Table 2 displays the odds ratios for the bivariate analyses between each of the explanatory variables and covariates and each of the nutrition outcomes. For the outcome of children’s ASF consumption, income, kebele, khat production ladder, and empowerment in leadership domain were associated with child ASF consumption at *p* < 0.2. The odds of reporting child ASF consumption in high-income households was 2.06 times that of low-income households. The odds of reporting ASF consumption in households in kebeles two, four, and five was 3.43 times that of households in kebeles one and three. The odds of reporting child ASF consumption in households with medium to high khat production ladder was 0.52 times that of households with low to no khat production ladder. The odds of reporting child ASF consumption in households with empowered women in the leadership domain was 2.23 times that of households with disempowered women in the leadership domain.

**Table 2:** Bivariate analyses between independent variables and child ASF consumption, dietary diversity, stunting, and underweight.

Independent variables	ASF consumption odds ratios (Min, Max)	Dietary diversity odds ratios (Min, Max)	Stunting odds ratios (Min, Max)	Underweight odds ratios (Min, Max)
Child sex^a^	1.16 (0.53, 2.54)	0.75 (0.19, 2.97)	1.52 (0.69, 3.37)	1.28 (0.53, 3.11)
Mother’s age^b^	1.32 (0.6, 2.9)	4.02^**^ (0.79, 20.49)	0.82 (0.37, 1.81)	1.32 (0.54, 3.2)
Mother’s education^c^	0.94 (0.38, 2.3)	1.35 (0.31, 5.84)	0.55 (0.21, 1.43)	0.74 (0.31, 1.79)
Assets^d^	1.75 (0.7, 3.86)	1.34 (0.34, 5.33)	0.61 (0.28, 1.36)	0.74 (0.31, 1.79)
Income^d^	2.06^*^ (0.93, 4.57)	1.40 (0.34, 5.58)	–	0.20 (0.65)
Kebele^e^	3.43^***^ (1.51, 7.79)	5.45^***^ (1.34, 23)	1.69 (0.75, 3.78)	2.36^**^ (0.96, 5.77)
TLU^b^	1.55 (0.7, 3.4)	6.81^**^ (0.82, 56.79)	0.71 (0.32, 1.58)	1.04 (0.43, 2.51)
Crop production as dominant livelihood^c^	1.92 (0.55, 6.7)	4.33^**^ (0.92, 20.38)	1.8 (0.56, 5.8)	5.89^***^ (1.73, 20.11)
Khat ladder^f^	0.52^*^ (0.23, 1.19)	0.28^**^ (0.06, 1.18)	1.59 (0.69, 3.66)	1.26 (0.50, 3.18)
Overall empowerment^c^	0.95 (0.43, 2.11)	2.31 (0.54, 10.83)	1.45 (0.65, 3.25)	1.16 (0.47, 2.86)
WEAI Domains				
Production^c^	0.96 (0.33, 2.82)	–	0.67 (0.23, 1.95)	1.11 (0.33, 3.8)
Resource credit^c^	0.57 (0.12, 2.69)	–	1.08 (0.23, 5.08)	1.12 (0.20, 6.14)
Income^c^	1.29 (0.3, 5.48)	–	0.68 (0.16, 2.88)	0.57 (0.13, 2.57)
Leadership^c^	2.23^*^ (0.82, 6.04)	0.85 (0.16, 4.46)	1.06 (0.39, 2.88)	0.80 (0.27, 2.36)
Time^c^	1.22 (0.56, 2.68)	1.87 (0.44, 7.94)	2.44^**^ (1.08, 5.54)	1.66 (0.67, 4.08)

a Reference group: male.

b Reference group: population with continuous values less than median.

c Reference group: population without the presence of the variable.

d Reference group: population with asset or income quartiles of 1 and 2.

e Reference group: kebeles 1 and 3 for ASF consumption; kebeles 1, 3, and 5 for dietary diversity; kebeles 2, 3, and 5 for stunting and underweight.

f Reference group: population with low to no involvement in khat production.

^*^p < 0.2, ^**^p < 0.1, ^***^p < 0.05 in chi-squared analyses. -Contingency table contained a value of 0 in at least one cell, so bivariate analysis was not performed. Chi-squared analyses performed; Fisher exact test performed if any cell in contingency table was less than or equal to 5. Values shown in each cell are unadjusted odds ratios (confidence intervals).

For the outcome of dietary diversity, mother’s age, kebele, TLU, crop production as dominant livelihood, and khat production ladder were associated with sufficient dietary diversity at *p* < 0.2. The odds of having sufficient child dietary diversity in households with an older mother (age above the median) was 4.02 times that of households with a younger mother. The odds of having sufficient child dietary diversity in households in kebeles two and four was 5.45 times that of households in kebeles one, three, and five. The odds of having sufficient child dietary diversity in households with a higher TLU (above the median) was 6.81 times that of households with a lower TLU. The odds of having sufficient child dietary diversity in households with crop production as their dominant livelihood was 4.33 times that of households who did not report crop production as their dominant livelihood. The odds of having sufficient child dietary diversity in households with medium to high khat production ladder was 0.28 times that of households with low to no khat production ladder.

For the outcome of stunting, only women’s empowerment in time domain was associated with stunting at *p* < 0.2. The odds of having a stunted child among households with empowered women in the time domain was 2.44 times that of households with disempowered women in the time domain. For the outcome of underweight, kebele and crop production as dominant livelihood were significantly associated with underweight at *p* < 0.2. The odds of having an underweight child among households in kebeles one and four was 2.36 times that of households in kebeles two, three, and five. Finally, the odds of having an underweight child among households with crop production as their dominant livelihood was 5.89 times that of households that did not report crop production as their dominant livelihood.

### 3.3. Multivariable regression models

Table 3 displays the results for the nested multivariable regression model for the outcome of ASF consumption. Kebele remained significant through all five models, such that the odds of child ASF consumption was significantly higher among households in kebeles two, four, and five than in households in kebeles one and three. Empowerment in the leadership domain was significant in model five, indicating that the odds of reporting child ASF consumption in households with empowered women in the leadership domain was 3.33 times that in households with disempowered women in the leadership domain.

**Table 3:** Nested multivariate regression models examining the relationship between demographic characteristics, livestock production, crop production, and women’s empowerment on ASF consumption.

Independent variables	Model 1	Model 2	Model 3	Model 4	Model 5
Income^a^	1.77 (0.77, 4.05)	1.66 (0.71, 3.91)	1.66 (0.71, 3.91)	1.83 (0.75, 4.48)	1.74 (0.69, 4.43)
Kebele^b^	3.17^**^ (1.38, 7.29)	3.15^**^ (1.37, 7.25)	3.14^**^ (1.3, 7.57)	2.68^*^ (1.03, 6.97)	3.06^*^ (1.1, 8.58)
TLU^c^		1.3 (0.55, 3.04)	1.3 (0.55, 3.04)	1.3 (0.56, 3.07)	1.19 (0.48, 2.97)
Crop production as dominant livelihood^d^			1.02 (0.26, 3.96)	0.98 (0.25, 3.84)	0.93 (0.22, 4.0)
Khat ladder^e^				0.67 (0.25, 1.8)	0.70 (0.24, 2.01)
WEAI Domains					
Production^d^					0.68 (0.15, 3.04)
Resource^d^					0.84 (0.13, 5.25)
Income^d^					0.86 (0.15, 5.0)
Leadership^d^					3.33^*^ (0.99, 11.15)
Time^d^					1.15 (0.46, 2.92)
Log-likelihood	−64.5	−64.4	−64.4	−64.1	−61.4
AIC	135.1	136.7	138.7	140.1	144.8
N	102	102	102	102	101

a Reference group: population with asset or income quartiles of 1 and 2.

b Reference group: kebeles 1 and 3.

c Reference group: population with continuous values less than median.

d Reference group: population without the presence of the variable.

e Reference group: population with low to no involvement in khat production.

^**^p < 0.01, ^*^p < 0.05, ^*p* < 0.10. Values shown in each cell are adjusted odds ratios.

Table 4 displays the results for the nested multivariate regression models for the outcome of stunting. In model five, the odds of having a stunted child in households with empowered women in the time domain was 2.68 times that in households with disempowered women in the time domain. Table 5 displays the results for the multivariate regression models for the outcome of underweight. Only kebele remained significant through all four models, indicating that the odds of an underweight child was significantly higher among households in kebeles one and four than in households in kebeles two, three, and five. Khat production ladder was not significant in any of the full models.

**Table 4:** Nested multivariate regression models examining the relationship between demographic characteristics, livestock production, crop production, and women’s empowerment on stunting.

Independent variables	Model 1	Model 2	Model 3	Model 5
TLU^a^	0.71 (0.32, 1.58)	0.7 (0.31, 1.55)	0.67 (0.3, 1.51)	0.59 (0.24, 1.42)
Crop production as dominant livelihood^b^		1.86 (0.57, 6.04)	2.24 (0.66, 7.62)	1.91 (0.54, 6.73)
Khat ladder^c^			1.83 (0.77, 4.36)	1.46 (0.58, 3.54)
WEAI Domains				
Production^b^				0.75 (0.2, 2.85)
Resources^b^				1.81 (0.33, 10.05)
Income^b^				1.22 (0.23, 6.42)
Leadership^b^				1.14 (0.35, 3.64)
Time^b^				2.68^*^ (1.07, 6.73)
Log-likelihood	−68.8	−68.2	−67.3	−64.3
AIC	141.5	142.4	142.5	146.5
N	102	102	102	101

a Reference group: population with continuous values less than median.

b Reference group: population without the presence of the variable.

c Reference group: population with little to no involvement in khat production.

^**^p < 0.01, ^*^p < 0.05, ^*p* < 0.10. Values shown in each cell are adjusted odds ratios.

**Table 5:** Nested multivariate regression models examining the relationship between demographic characteristics, livestock production, crop production, and women’s empowerment on underweight.

Independent variables	Model 1	Model 2	Model 3	Model 4
Mother’s Education^a^	0.44 (0.14, 1.37)	0.43 (0.13, 1.37)	0.41 (0.13, 1.34)	0.43 (0.13, 1.45)
Kebele^b^	2.95^*^ (1.16, 7.54)	2.96^*^ (1.16, 7.57)	3.32^*^ (1.17, 9.44)	3.38^*^ (1.07, 10.66)
TLU^c^		1.09 (0.43, 2.77)	1.11 (0.43, 2.83)	1.22 (0.45, 3.36)
Khat ladder^d^			0.76 (0.26, 2.19)	0.75 (0.24, 2.27)
WEAI Domains				
Production^a^				1.73 (0.33, 9.0)
Resources^a^				1.48 (0.21, 10.24)
Income^a^				0.58 (0.10, 3.42)
Leadership^a^				0.50 (0.14, 1.79)
Time^a^				1.37 (0.46, 4.03)
Log-likelihood	−55.1	−55.1	−55.0	−53.7
AIC	116.3	118.2	120.0	127.6
N	100	100	100	99

a Reference group: population without the presence of the variable.

b Reference group: kebeles 2, 3, and 5.

c Reference group: population with continuous values less than median.

d Reference group: population with low to no involvement in khat production.

^**^p < 0.01, ^*^p < 0.05, ^*p* < 0.10. Values shown in each cell are adjusted odds ratios.

## 4. Discussion

This study aimed to explore the relationship between women’s empowerment and child nutritional outcomes in rural smallholder farming communities where cash-crop production of khat has rapidly increased. No clear pattern of livelihood and empowerment emerges from these data to explain nutritional outcomes in children; however, important associations have been identified and contribute to a growing body of literature around women’s empowerment and nutritional outcomes.

First, khat production and TLU were not significantly associated with any of the child nutrition outcomes in the full models. Some evidence indicates that households that are engaged in smallholder production may need to meet a certain income or production threshold before production begins to significantly benefit child ASF consumption (36, 37). Some households may not keep livestock for routine ASF consumption and instead use livestock as direct or indirect income sources. Eastern Ethiopia is characterized by high population pressure and small, fragmented land systems, contributing to limited agricultural inputs and low productivity, which is exacerbated by climate change affecting the region (38, 39). Furthermore, while some crop production may increase food availability, this may not translate to improved nutritional outcomes. Interestingly, these findings also indicate that these households may not significantly rely on livestock production or khat production directly for child nutrition.

Access to markets was not included in this analysis; however, the implication of household access to markets on nutrition is important to consider. Smallholder farming households that are closer to markets may be better integrated into the cash economy and may be more likely to sell their livestock or agricultural products for income instead of consuming their own products. Markets can also enable access to other resources and foods that contribute to nutrition and dietary diversity, complementing household’s own production with other foods that may diversify children’s diets (15, 40–42). This mix of subsistence and market and/or commercialized-oriented production adds to the complexity of assessing the relationship between smallholder production and dietary outcomes. The role of women’s nutrition knowledge also plays a necessary, albeit not sufficient, role in improving child nutrition, as caregivers who are more knowledgeable about diet and nutrition are better equipped to make informed decisions about high quality diets (43). Indeed, research has demonstrated that improving nutrition knowledge among women in low education settings is positively related to children’s diets (44–47).

Production diversity is another potential strategy to improve the nutrition of smallholder households. Diversifying livestock and crop production may improve household dietary diversity and can also contribute to a more stable income by buffering households from market risks, price volatility, and production output variability (48). Khat is one way smallholder households in eastern Ethiopia may diversify production and begin to commercialize parts of household production. While the khat production ladder was not significantly associated with the odds of any of the nutrition outcomes in this analysis, khat production may still play an important role in households’ livelihood in other ways. Formative research from the CAGED study found that khat has become increasingly commercialized over the past 10–15 years in Haramaya, Ethiopia (18). The implications of khat production and commercialization on child nutrition in Ethiopia are complex. While some khat is consumed by households, households may rely on income from the sale of khat to purchase food and other goods.

Khat prices, however, can fluctuate, making it difficult for smallholder farmers to depend on khat as a reliable source of income. Furthermore, some research indicates that women are heavily involved in the production and sale of khat (25). This may contribute to women’s empowerment in meaningful ways, such as increasing decision-making over production and sale of crops, but it may also increase the work intensity of women in households with labor scarcity. There is little research on women’s involvement in the khat value chain in Ethiopia, and how women’s (dis)empowerment has shifted in agriculture and livestock production. Future research focusing on women’s roles, responsibilities, and agency in the context of transitioning livelihoods from subsistence farming to cash crop production, transport, and selling in Ethiopia is needed to better inform the pathway linking women’s empowerment to child nutrition and to inform future interventions targeting agriculture and livestock production and child nutrition in Ethiopia.

The overall women’s empowerment (5DE) score was not associated with any of the child nutrition outcomes in the full models. However, when the 5DE is disaggregated by domain, the domains of women’s empowerment in leadership and time were significantly associated with children’s ASF consumption and stunting, respectively. Women who were empowered in the leadership domain were significantly more likely to report having children who consumed ASF compared to women who were not empowered in this domain. Empowerment in the leadership domain may correspond with a woman’s self-efficacy in making health-related decisions and advocating for herself and her family. Group membership, a sub-domain of the leadership domain, may also contribute to building social networks with others in a community, facilitating access to resources, knowledge, and social support (49, 50). Furthermore, membership in certain groups, such as agricultural or economic groups, may indirectly benefit women’s participation in decision-making by providing a platform for discussions and strategic partnerships (49). The association between women’s empowerment in leadership and child ASF consumption has important implications on livestock programs that aim to improve nutrition; specifically, programs may want to leverage this pathway by focusing on approaches that cultivate female leaders.

Interestingly, in contrary to expected results, women’s empowerment in the time domain was significantly associated with increased odds of having a child who was stunted, compared to women who were not empowered in this domain. The time domain consists of a woman’s time allocation to productive and domestic tasks, as well as the level of satisfaction with her time for leisure activities. Quisumbing et al. (51) discuss tradeoffs among dimensions of empowerment and nutrition outcomes, noting that not all empowerment domains may be positively correlated with better nutrition in every context. Women may increase their workload to provide more and higher quality food to their households, but the increased workload may translate to less time for childcare and increased energy expenditure (51). Additionally, women’s use of time for different activities, such as domestic versus agricultural/livestock activities, may have different contributions to child nutrition outcomes, but the relationship between specific time use activities and child nutrition outcomes is complex.

Nutritional implications of increased time burdens of women are complex, as households respond to increased time burdens and workloads in different ways (52). Analysis of time use data from several countries in sub-Saharan Africa and Asia found that more time spent on domestic activities and cooking was positively correlated with child dietary diversity in poor households (53). More time spent in agriculture had differing effects on child nutrition for poor vs. nonpoor women, such that more women’s time in agriculture was positively associated with children’s minimum acceptable diet among poor women, while the inverse was true for children of nonpoor women, demonstrating how existing assets and income may be important for how women are able to allocate time and the effects that increased time burdens have on nutritional outcomes.

In the context of this study, women khat sellers may have increased time burdens but may also have more income to purchase food for their household as well as market access, whereas poorer women who are not engaged in khat selling may have more time allocated to domestic activities but may not have the resources to access and purchase diversified food. If a woman is empowered in the time domain (such that she has a high level of satisfaction with leisure time and an appropriate allocation of time to productive and domestic tasks), it is possible, then, that she may not have enough time devoted to productive tasks that produce benefits for the household. While having an excessive workload for women is not ideal, it may contribute to child nutrition outcomes in unexpected ways, such as more productive crops and livestock or increased income generation for the household.

There are potential benefits and drawbacks for child nutrition when shifting women’s time from domestic to agricultural or livestock activities, and outcomes may even differ for households whose livelihoods are livestock-dominant versus crop-dominant (54). Additionally, given that the households in this study sample are smallholder farming households, seasonality may be another important differentiating factor for child nutrition outcomes. There may be seasonal variation in time allocation and child nutrition outcomes (55). The results from this study support the idea that empowerment domains may have different impacts on child nutrition outcomes, and contextual factors and location may also further drive which domains have the most impact on these outcomes (56, 57).

There are several limitations with this study. The cross-sectional nature of these data limits the conclusions that can be made about the associations between livelihoods, empowerment, and child nutrition outcomes. Children from families where livestock that has just been acquired, for instance, may not have had the time to benefit from that livestock. Additionally, this study did not collect WEAI data for men; thus, this study did not include a measure of gender parity for the women and men in the study sample.

## 5. Conclusion

As low and middle-income countries develop, livelihood strategies will continue to change, and the important role of women’s empowerment on child nutritional outcomes may shift, accordingly. While no associations between khat production and child nutrition were identified, khat production, and, more broadly, cash crop production, remain important livelihood strategies that may be contributing to shifts in livelihoods and gender dynamics in smallholder farming households in Ethiopia and other low-and-middle-income countries. This study identified limited associations between women’s empowerment and child nutrition, underscoring the need to understand local context and the myriad, nuanced, intersecting ways that efforts to target specific domains of women’s empowerment may facilitate – or hinder – nutritional improvements.

## Data availability statement

The original contributions presented in the study are publicly available. This data can be found at: https://dataverse.harvard.edu/dataset.xhtml?persistentId=doi:10.7910/DVN/CPCK91.

## Ethics statement

The studies involving human participants were reviewed and approved by Haramaya University Institutional Health Ethics Research Review Committee (Ref. no. IHRERC/152/2018), Ethiopia National Research Ethics Review Committee (Ref. no. MoST/3-10/168/2018), Institutional Review Board at the University of Florida (Ref. no. 201703252), and Washington University School of Medicine (Protocol no. 201806021). Written informed consent to participate in this study was provided by the participants’ legal guardian/next of kin.

## Author contributions

AH, SM, JH, WG, MM, GR, and YY conceived and designed the study. IA, JA, IU, and KR collected the data. AI and KR supervised the data collection and managed the research activities. NS, DC, and XL cleaned and processed the data. KM, NS, DC, and XL analyzed the data, with supervision from AH, SM, and YY. KM and NS wrote the first draft of the manuscript. AR and AC wrote sections of the manuscript. All authors contributed to the article and approved the submitted version.

## Funding

This work was supported, in whole or in part, by the Bill & Melinda Gates Foundation [OPP11755487]. Under the grant conditions of the Foundation, a Creative Commons Attribution 4.0 Generic License has already been assigned to the Author Accepted Manuscript version that might arise from this submission. The University of Florida was funded by the Bill & Melinda Gates Foundation to research and address food insecurity issues in Ethiopia and Burkina Faso through the project Equip—Strengthening Smallholder Livestock Systems for the Future. These funds are administered by the Feed the Future Innovation Lab for Livestock Systems, which was established by funding from the United States Agency for International Development (USAID) and is co-led by the University of Florida’s Institute of Food and Agricultural Sciences and the International Livestock Research Institute. Support for the Feed the Future Innovation Lab for Livestock Systems is made possible by the generous support of the American people through USAID. The contents are the responsibility of the authors and do not necessarily reflect the views of USAID or the United States Government.

## Conflict of interest

The authors declare that the research was conducted in the absence of any commercial or financial relationships that could be construed as a potential conflict of interest.

## Publisher’s note

All claims expressed in this article are solely those of the authors and do not necessarily represent those of their affiliated organizations, or those of the publisher, the editors and the reviewers. Any product that may be evaluated in this article, or claim that may be made by its manufacturer, is not guaranteed or endorsed by the publisher.
